# Reducing and meta-analysing estimates from distributed lag non-linear models

**DOI:** 10.1186/1471-2288-13-1

**Published:** 2013-01-09

**Authors:** Antonio Gasparrini, Ben Armstrong

**Affiliations:** 1 Department of Medical Statistics, London School of Hygiene and Tropical Medicine, London, UK; 2 Department of Social and Environmental Health Research, London School of Hygiene and Tropical Medicine, London, UK

**Keywords:** Distributed lag models, Multivariate meta-analysis, Two-stage analysis, Time series

## Abstract

**Background:**

The two-stage time series design represents a powerful analytical tool in environmental epidemiology. Recently, models for both stages have been extended with the development of distributed lag non-linear models (DLNMs), a methodology for investigating simultaneously non-linear and lagged relationships, and multivariate meta-analysis, a methodology to pool estimates of multi-parameter associations. However, the application of both methods in two-stage analyses is prevented by the high-dimensional definition of DLNMs.

**Methods:**

In this contribution we propose a method to synthesize DLNMs to simpler summaries, expressed by a reduced set of parameters of one-dimensional functions, which are compatible with current multivariate meta-analytical techniques. The methodology and modelling framework are implemented in R through the packages dlnm and mvmeta.

**Results:**

As an illustrative application, the method is adopted for the two-stage time series analysis of temperature-mortality associations using data from 10 regions in England and Wales. R code and data are available as supplementary online material.

**Discussion and Conclusions:**

The methodology proposed here extends the use of DLNMs in two-stage analyses, obtaining meta-analytical estimates of easily interpretable summaries from complex non-linear and delayed associations. The approach relaxes the assumptions and avoids simplifications required by simpler modelling approaches.

## Background

Research on the health effects of environmental stressors, such as air pollution and temperature, often relies on time series analysis using data from multiple locations, usually cities [1,2]. The analytical design adopted in this setting is commonly based on two-stage procedures, where location-specific exposure-response relationships are estimated through a regression model in the first stage, and these estimates are then combined through meta-analysis in the second stage [3].

Recently, the first-stage modelling approaches have been extended with the introduction of *distributed lag non-linear models* (DLNMs) [4,5], a methodology to describe simultaneously non-linear and delayed dependencies. This modelling class is based on the definition of a *cross-basis*, a bi-dimensional space of functions describing the association along the spaces of predictor and lags. The cross-basis is specified by the choice of two basis, one for each dimension, among a set of possible options such as splines, polynomials, or step functions. Concurrently, developments have been proposed also for the second stage. In particular, techniques based on multivariate meta-analysis have been used to combine estimates of associations defined by multiple parameters, and applied for either non-linear [6-8] or lagged dependencies [8-11]. We recently provided an overview of the use of multivariate meta-analysis in this setting [12].

In this contribution we propose a method to reduce estimates from DLNMs to summaries defined in only one dimension of predictor or lags, re-expressing the fit in terms of reduced parameters for the related uni-dimensional basis functions. This step decreases the number of parameters to be pooled in the second stage, offering a method to meta-analyse estimates from richly parameterized non-linear and delayed exposure-response relationships.

In the next section, we provide a brief recap of the algebraic development of DLNMs and multivariate meta-analysis, and then describe the main statistical development, establishing a method to reduce the fit of a DLNM to summaries expressed in a single dimension. A motivating example with an analysis of the relationship between temperature and all-cause mortality is used throughout the paper to illustrate the statistical framework. We finally note some limitations and indicate future directions for research. Supplementary online material provides information on algebraic notation and software (Additional file 1), and also includes the R code and data to entirely reproduce the results in the example (Additional files 2– 3).

## Methods

The two-stage time series design can be applied to series of observations collected at each time *t*, with *t* = 1,…,*N*_*i*_, in each location *i*, with *i* = 1,…,*m*. First-stage regression models are fitted to each series of *N*_*i*_ observations, obtaining location-specific estimates of the association of interest. These estimates are then pooled across locations in the second stage, with the aim to estimate an average exposure-response relationship and inspect heterogeneity across locations.

### An illustrative example

As an illustration, we describe an analysis of the relationship between temperature and all-cause mortality using daily series of *N*_*i*_ = *N* = 5113 observations from each of the *m* = 10 regions in England and Wales, in the period 1993–2006. Further details on the dataset were previously provided [13,14]. The example is used throughout the paper to describe the steps of the modelling framework and introduce the new methodological development. Specifically, the relationship is flexibly estimated in each region in the first-stage analysis using DLNMs, and then pooled in the second stage through multivariate meta-analysis. The example aims to demonstrate how results from DLNMs are summarized in an analysis of a single region, and then how these reduced summaries can be combined across regions. Also, we illustrate a comparison with simpler modelling approaches. Modelling choices are dictated by illustrative purposes, and the results are not meant to provide substantive evidence on the association.

### Distributed lag non-linear models

The DLNM framework has been extensively described [5]. Here we provide a brief overview to facilitate the new development, illustrated later. In particular, we will focus on the bi-dimensional structure of this class of models, represented by the two sets of basis functions applied to derive the parameterization. Following the original paper, we first generalize the idea of simple distributed lag models (DLMs) and then introduce the non-linear extension.

#### The DLNM modelling class

Distributed lag linear and non-linear models are expressed through a lag-basis and cross-basis function *s*(*x*_*t*_), respectively, of the *N*-length exposure series **x** =[*x*_1_,…,*x*_*t*_,…,*x*_*N*_]^T^. The definition of *s*(*x*_*t*_) first requires the derivation of the *N* × (*L* + 1) matrix **Q** of lagged exposures, so that **q**_*t*·_ =[*x*_*t*_,…,*x*_*t*−*ℓ*_,…,*x*_*t*−*L*_]^T^. This step indirectly characterizes the new lag dimension identified by the vector ***ℓ*** =[0,…,*ℓ*,…,*L*]^T^, with *L* as maximum lag. Now, choosing a first basis with dimension *v*_*ℓ*_ to represent the association along the new lag space, we can compute a (*L* + 1) × *v*_*ℓ*_ basis matrix **C** by applying the related functions to ***ℓ***. A compact and general definition of the lag-basis function *s*(*x*_*t*_) for DLMs is given by:

(1) $$s(x_{t};\eta )=\overset{v_{\ell }}{\underset{k=1}{\sum }}q_{t\cdot }^{T}c_{\cdot k}\eta_{k}=q_{t\cdot }^{T}C\eta =w_{t\cdot }^{T}\eta \text{,}$$

where different models are specified with different choices of the basis to derive **C**. The transformed variables in **W** = **QC** can be included in the design matrix of the first-stage regression model, in order to estimate the *v*_*ℓ*_-length parameter vector ***η***, with **C*η***representing the lag-specific contributions.

The non-linear extension to DLNMs requires the choice of a second basis with dimension *v*_*x*_ to model the relationship along the space of the predictor *x*, obtaining the *N* × *v*_*x*_ basis matrix **Z** from the application of the related functions to **x**. Applied together with the transformation which defines the matrix of lagged exposures **Q** above, this step produces a three-dimensional *N* × *v*_*x*_ × (*L* + 1) array $\dot{R}$. The parameterization of the cross-basis function *s*(*x*_*t*_) for DLNMs is then given by:

(2) $$s(x_{t};\eta )=\overset{v_{x}}{\underset{j=1}{\sum }}\overset{v_{\ell }}{\underset{k=1}{\sum }}r_{\text{tj}\cdot }^{T}c_{\cdot k}\eta_{\text{jk}}=w_{t\cdot }^{T}\eta \text{.}$$

The simpler lag-basis for DLMs in (1) is a special case of the more complex cross-basis for DLNMs in (2). These models may be fitted through common regression techniques with the inclusion of cross-basis matrix **W** in the design matrix. The vector $\hat{\eta }$ of estimated parameters of the cross-basis function in (2) represents a simultaneously non-linear and lagged dependency, and its length *v*_*x*_ × *v*_*ℓ*_ is equal to the product of the dimensions of the bases for the two spaces. In completely parametric models as those described here, this dimensionality is directly associated with the notion of *degrees of freedom* (*df*), related to the flexibility of the function and the smoothness of the estimated dependency. In spite of the relatively complex algebraic definition in (2), DLNMs are solely specified by the choice of the two bases for deriving the matrices **Z** and **C**. The software implementation of this methodology in the R package dlnm has been previously described [15].

#### Summarizing the results from a DLNM

Fitted bi-dimensional cross-basis functions from DLNMs can be interpreted by deriving predictions over a grid of predictor and lag values, usually computed relative to a reference predictor value. As a first example, we show the results of a single-location analysis, using data from the North-East region of England. The temperature-mortality relationship is modelled through the same cross-basis used for the full two-stage analysis illustrated later, composed by two B-spline bases.

**Figure 1:**
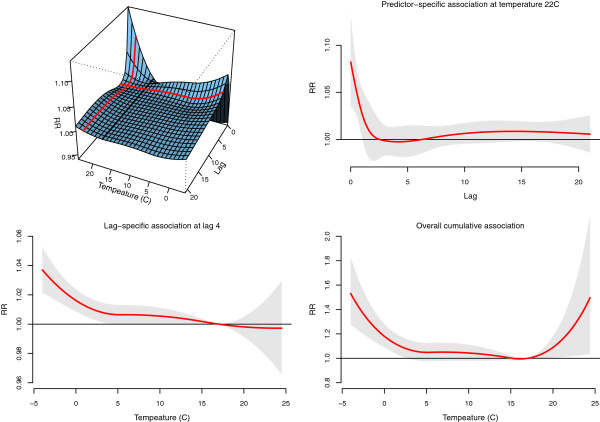
**Temperature-mortality association in the North-East region of England, 1993–2006.***Top-left*: 3-D graph (with black reference line at 17°C). *Top-right*: predictor-specific summary at 22°C (red line parallel to the reference in the 3-D graph). *Bottom-left*: lag-specific summary at lag 4 (red line perpendicular to the reference in the 3-D graph). *Bottom-right*: overall cumulative summary. The 95%CI are reported as grey areas.

The results are shown in Figure 1. The top-left panel displays the bi-dimensional surface of the fitted relative risk (RR) in a 3-D graph, predicted for the grid of temperature and lag values, with a reference black line corresponding to the centering value of the basis for the predictor space (here 17°C). Similarly to previous analyses, the figure suggests an immediate increase in risk for high temperature, and a more delayed but protracted effect for low temperature.

This bi-dimensional representation contains details not relevant for some interpretative purposes, and does not easily allow presentation of confidence intervals. The analysis therefore commonly focuses on three specific uni-dimensional *summaries* of the association, also illustrated in Figure 1. First, a *predictor-specific* summary association at a given predictor value *x*_0_ can be defined along the lag space. As an example, this is reproduced in the top-right panel for temperature *x*_0_ = 22°C, together with 95% confidence intervals (CI), and corresponds to the red line parallel to the reference in the 3-D graph. Second, similarly, a *lag-specific* summary association at a given lag value *ℓ*_0_ can be defined along the predictor space. This is shown in the bottom-left panel for lag *ℓ*_0_ = 4, and coincides with the red line in the 3-D graph perpendicular to the reference. Third, the sum of the lag-specific contributions provides the *overall cumulative* association, showed in the bottom-right panel of Figure 1. This last summary offers an estimate of the net effect associated with a given exposure cumulated over the lag period *L*, and is usually the focus of the analysis.

### Multivariate meta-analysis

The framework of multivariate meta-analysis has been previously described [16], and its application for combining estimates of multi-parameter associations has been recently illustrated [12]. We offer a brief summary here, firstly illustrating the second-stage multivariate meta-analytical model and then discussing its limitation for pooling DLNMs.

#### The multivariate extension of meta-analysis

Specification of the model assumes that a *k*-dimensional set of outcome parameters ${\hat{\eta }}_{i}$ and associated *k* × *k* (co)variance matrix **S**_*i*_ have been estimated in each of the *i* = 1,…,*m*studies. In the application for two-stage time series analysis, these outcome parameters represent regression coefficients from the first stage, while the term study refers here to the first-stage analysis in each location. The description below illustrates a random-effects multivariate meta-regression model, where fixed-effects models or simple meta-analysis treated as special cases. The model for location *i* is defined as:

(3) $${\hat{\eta }}_{i}∼N\left(\right)U_{i}\beta \;,\;S_{i}+\Psi ))\text{,}$$

where the location-specific estimated outcome parameters ${\hat{\eta }}_{i}$ are assumed to follow a *k*-dimensional multivariate normal distribution. The *k*×*kp* block-diagonal matrix $U_{i}=I_{\left(k\right)}⊗u_{i}^{T}$ is the Kronecker expansion of the location-specific vector **u**_*i*_ = *u*_1_,…,*u*_*p*_^T^ of meta-variables. The matrices **Ψ** and **S**_*i*_ represent the between and within-location (co)variance matrices, respectively. This multivariate meta-regression model is applied to estimate the parameter vectors ***β*** and ***ξ***. The former represents the *kp* second-stage coefficients defining how the *p* meta-variables are associated with each of the true *k* first-stage coefficients in ***η***_*i*_. The vector ***ξ*** includes a set of parameters which uniquely define the between-location (co)variance matrix **Ψ**, depending on the chosen structure for this matrix. In fixed effects models no additional variability beyond the estimation error in the first-stage model is assumed for ${\hat{\eta }}_{i}$, and **Σ**_*i*_ = **S**_*i*_. In multivariate meta-analysis with no meta-variable, **U** = **I**_(*k*)_ and ***β*** = ***η***, the vector of average parameters. The development in (3) can be considered as a special case of multivariate linear mixed model [17], where the within-city (co)variance is assumed known. Among alternative estimation methods, such as Bayesian [18] and multivariate extensions of the method of moments [19], we privilege here likelihood-based approaches [20,21]. Methods to derive tests and confidence intervals, fit statistics and (best-linear unbiased) predictions have been previously developed within the linear mixed models framework for the application in this setting, together with a description of the software implementation in the R package mvmeta[12].

#### Limitations of multivariate meta-analysis

In theory, the *m* sets of estimated first-stage coefficients ${\hat{\eta }}_{i}$ of the cross-basis obtained from DLNMs in (2) can be meta-analysed using (3), producing an population-averaged three-dimensional effect surface across locations, optionally conditional on meta-variables in multivariate meta-regression. However, as anticipated above, the definition of DLNMs in (2) requires *k* = *v*_*x*_ × *v*_*ℓ*_ parameters ${\hat{\eta }}_{i}$ for the cross-basis. For models specified by even moderately complex bases in each space, the number of parameters becomes so high that the optimization routine for multivariate meta-analysis is computationally impractical. This is particularly relevant for the (co)variance terms in ***ξ*** defining the true between-location variability, composed by *k*(*k* + 1)/2 parameters for an unstructured matrix **Ψ**.

This limitation is one of the main reasons which have prevented so far the full application of DLNMs in two-stage analysis. The modelling approach has often required the simplification of the first-stage model, for the second-stage multivariate meta-analysis to be feasible. For example, investigators have assumed a linear relationship in the dimension of the predictor [8-11], or computed a simple exposure moving average for the lag space [6-8]. We previously adopted the same limited approach [12]. The development of methods to derive meta-analytical estimates from full DLNMs would offer a great deal of flexibility in the investigation of complex exposure-response dependencies.

### Reducing DLNMs

Predictions from DLNMs as those shown in Figure 1 are obtained by selecting the grid of exposure and lag values defined as **x**_[*p*]_ and ***ℓ***_[*p*]_, respectively. Details on the algebraic development are given elsewhere [5] [sections 4.2–4.3]. Briefly, predictions are computed by deriving matrices **Z**_[*p*]_ and **C**_[*p*]_ from **x**_[*p*]_ and ***ℓ***_[*p*]_, respectively, through the application of the same basis functions used for estimation in (2). The bi-dimensional predicted relationship in location *i* is expressed by the full set of estimated parameters in ${\hat{\eta }}_{i}$ and quantities derived by **Z**_[*p*]_ and **C**_[*p*]_. However, the specific summaries described in the previous section are defined only on the single dimension of predictor (lag-specific and overall cumulative associations, bottom panels of Figure 1) or lag (predictor-specific association, top-right panel of Figure 1). The idea is to re-parameterize these summaries in terms of the related uni-dimensional basis **Z**_[*p*]_ or **C**_[*p*]_ for predictor or lags only, respectively, and sets of reduced in coefficients ${\hat{\theta }}_{i}$. Dimensionality of the functions expressing such summaries therefore decreases from *v*_*x*_ × *v*_*ℓ*_, corresponding to the length of vector ${\hat{\eta }}_{i}$ in the original parameterization, to *v*_*x*_ or *v*_*ℓ*_ only, corresponding to the length of new sets of reduced parameters ${\hat{\theta }}_{i}$.

The definition of the reduced parameters depends on the specific summary among those listed above. They can be obtained by applying a related dimension-reducing matrix **M**, expressed as:

(4a) $$\begin{array}{ll} & M_{\left[x_{0}\right]}=I_{\left(v_{\ell }\right)}⊗z_{\left[x_{0}\right]}^{T}\; \end{array}$$

(4b) $$\begin{array}{ll} & M_{\left[\ell_{0}\right]}=c_{\left[\ell_{0}\right]}^{T}⊗I_{\left(v_{x}\right)}\; \end{array}$$

(4c) $$\begin{array}{ll} & M_{\left[c\right]}=1_{\left(L+1\right)}^{T}C⊗I_{\left(v_{x}\right)}\; \end{array}$$

for predictor-specific summary association at *x*_0_, for for lag-specific summary association at *ℓ*_0_, and for overall cumulative summary association, respectively. Here $z_{\left[x_{0}\right]}$ and $c_{\left[\ell_{0}\right]}$ are the vectors of transformed values of *x*_0_and *ℓ*_0_ obtained by the application of the sets of basis functions for predictor and lags, respectively. The reduced parameter vector ${\hat{\theta }}_{i}$ and associated (co)variance matrix $V({\hat{\theta }}_{i})$ are then obtained by:

(5) $$\begin{array}{cc} & {\hat{\theta }}_{[j]i}=M_{\left[j\right]}{\hat{\eta }}_{i}\\ \;V({\hat{\theta }}_{[j]i}) & =M_{\left[j\right]}V({\hat{\eta }}_{i})M_{\left[j\right]}^{T}\text{,} \end{array}$$

with *j* ∈ {*x*_0_,*ℓ*_0_,*c*}. The predictor-specific association at *x*_0_, defined on the lag space for values in ***ℓ***_[*p*]_, is expressed by $C_{\left[p\right]}{\hat{\theta }}_{[x_{0}]i}$, with standard errors provided by the square root of $C_{\left[p\right]}V({\hat{\theta }}_{[x_{0}]i})C_{\left[p\right]}^{T}$. The lag-specific association at *ℓ*_0_ and the overall cumulative association, defined on the predictor dimension for values in **x**_[*p*]*i*_, are then obtained by $Z_{\left[p\right]}{\hat{\theta }}_{[\ell_{0}]i}$ and $Z_{\left[p\right]}{\hat{\theta }}_{[c]i}$, respectively, with computation for standard errors as above. The number of coefficients defining these summaries, reduced from *v*_*x*_ × *v*_*ℓ*_ to *v*_*ℓ*_ or *v*_*x*_ only, is usually compatible with the application of standard multivariate meta-analysis techniques, which can now be used to combine the estimates of these summaries from DLNMs in two-stage analyses. In addition, the derivation in (4)–(5) simplifies the algebra for DLNM predictions originally provided [5] [Section 4.3]. The dimension reduction comes at the price of loss of information about the association on one of the two dimensions, as the rank-deficiency of **M** does not allow reversing the reduction applied in (5).

## Results

The analysis is now extended to the full set of 10 regions, with the aim to produce pooled estimates of the overall cumulative association, and to compare the results with those obtained by simpler approaches, applying a moving average to the daily exposure series. Also, we investigate the lag structure for exposure to cold and hot temperatures through predictor-specific estimates. Finally, we assess heterogeneity and then the role of meta-variables through multivariate meta-regression.

### Modelling strategy

The first-stage region-specific model is specified by adopting a standard analytical approach for time series environmental data [1,3,4]. In each region, we fit a common generalized linear model for the quasi-Poisson family to the series of all-cause mortality counts. The model includes the cross-basis for daily mean temperature, a natural cubic spline of time with 10 *df* / year to control for the long-term and seasonal variation, and indicator variables for day of the week.

In the main first-stage model, the temperature-mortality association is estimated by a flexible cross-basis defined by a quadratic B-spline for the space of temperature, centered at 17°C, and a natural cubic B-spline with intercept for the space of lags, with maximum lag *L* = 21. We place two internal knots at equally spaced values along temperature (5.3°C and 15.1°C) and three internal knots at equally-spaced log-values of lag (1.0, 2.8 and 7.6), with boundary knots at −4.4°C and 24.9°C, and 0 and 21 lags, respectively. These choices define spline bases with dimensions *v*_*x*_ = 4 and *v*_*ℓ*_ = 5 for temperature and lag spaces, respectively. The same specification was previously applied for the single-region analysis.

The set of *v*_*x*_ × *v*_*ℓ*_ = 20 coefficients of the cross-basis variables with associated (co)variance matrices, estimated in each region, are then reduced. Specifically, for region *i* we derive the vector ${\hat{\theta }}_{[c]i}$ with 4 reduced parameters of the quadratic B-spline of temperature **Z**_[*p*]_ for the overall cumulative summary association, and two vectors ${\hat{\theta }}_{[0]i}$ and ${\hat{\theta }}_{[22]i}$ with sets of 5 reduced parameters of the natural cubic B-spline of lags **C**_[*p*]_ for predictor-specific summary associations at 0°C and 22°C. These temperatures correspond approximately to the 1^st^ and 99^th^ of the pooled temperature distribution, respectively. These effects along lags are interpreted using the reference of 17°C.

For comparison with methods not requiring dimensionality reduction, in two alternative first-stage models we simplify the lag structure by fitting one-dimensional splines to the moving average of the temperature series over lag 0–3 and 0–21, respectively. Such moving average models have been commonly used in weather and air pollution epidemiology [4,10,22]. These alternatives can be described as DLNMs including cross-bases with a constant function to represent the relationship in the lag space, while keeping the same quadratic B-spline for the space of the predictor, as described for the main model above. In these simplified models, the dimension of fitted relationship does not need to be reduced. In fact, the application of the reduction method returns the original *v*_*x*_ × *v*_*ℓ*_ = 4 × 1 = 4 parameters re-scaled by the number of lags, giving a dimension-reducing matrix **M**_[*c*]_, as described in (4c), composed in this case by a diagonal matrix with entries corresponding to a constant equal to the number of lags.

The coefficients for each of the three summary associations from the main model are estimated in the 10 regions and then independently included as outcomes in three multivariate meta-analytical second-stage models. The ten estimated sets of coefficients from the two alternative models (equivalent to the overall cumulative summary) were directly meta-analysed. All the second-stage models are fitted here through restricted maximum likelihood (REML) using the R package mvmeta. We first derive an estimate of the pooled relationship through multivariate meta-analysis, and then extend the results showing an example of multivariate meta-regression which includes population-averaged regional latitude as a meta-variable. The effect of latitude is displayed by predicting the averaged temperature-mortality associations for the 25^th^ and 75^th^ percentiles of its distribution, using the same baseline reference of 17°C. The significance of such an effect is assessed through a Wald test, given a likelihood ratio test cannot be applied to compare model fitted with REML and different fixed-effects structures [12].

### Two-stage analysis

**Figure 2:**
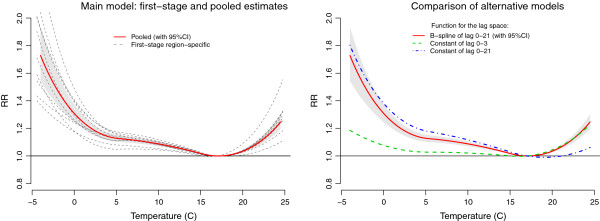
**Pooled overall cumulative temperature-mortality association in 10 regions of England and Wales, 1993–2006.***Left panel*: first-stage region-specific and pooled (95%CI as grey area) summaries from the main model. *Right panel*: comparison of alternative models.

The overall temperature-mortality associations in the 10 regions of England and Wales are illustrated in Figure 2. The left panel shows the regions-specific summary associations from the first stage, together with the pooled average from multivariate meta-analysis, as predicted by the main flexible model. Regions-specific estimates show similar curves, although some variability exists, in particular at the extremes. Consistently with previous findings, the pooled curve suggests an increase in relative risk (RR) for both cold and hot temperatures, although less pronounced for the latter, and with a steeper increase for extreme when compared to mild cold. The average point of minimum mortality is at 17.1°C, corresponding approximately to the 90^th^ percentile of the pooled temperature distribution. The multivariate Cochran Q test for heterogeneity is highly significant (*p*-value < 0.001), and the related *I*^2^ statistic indicates that 63.7% of the variability is due to true heterogeneity between regions.

The right panel of Figure 2 illustrates the comparison with the two alternative simpler models. We see that the association based on the 0–21 lag moving average temperature approximates that based on a flexible DLNM in the cold range, but completely misses the heat effect. The reverse is true for the association based on the 0–3 lag moving average temperature.

**Figure 3:**
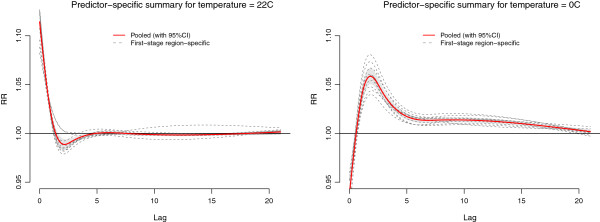
**Pooled predictor-specific temperature-mortality association in 10 regions of England and Wales, 1993–2006.** First-stage region-specific and pooled (95%CI as grey area) summaries at 22°C (left panel) and 0°C (right panel). Reference at 17°C.

Figure 3 depicts the pooled estimate from the main model for predictor-specific summary associations at 22°C and 0°C, with the same reference of 17°C, as predicted by the two sets of *v*_*ℓ*_ = 5 reduced coefficients. Consistently with previous research, the effect of hot temperature is immediate and disappears after 1–2 days, while cold temperatures are associated with mortality for a long lag period, after an initial protective effect. This complex lag pattern can explain the different results provided by the less flexible alternative models. The pooled overall RR estimated by the main model, cumulated along lags for these specific summaries and reported graphically in Figure 2 (left panel), are 1.101 (95%CI: 1.078–1.124) for 22°C and and 1.308 (95%CI: 1.245–1.375) for 0°C, respectively. The Cochran Q test is significant for the lag curve at 0°C (*p*-value < 0.001), but not for that at 22°C (*p*-value = 0.178), with an *I*^2^ of 63.4% and 16.0%, respectively.

It is interesting to note that the second-stage multivariate meta-analytical model for the predictor-specific summary associations at 22°C estimates perfectly correlated random components, with between-study correlations equal to −1 or 1. This is a known phenomenon in multivariate meta-analysis, frequently occuring in the presence of a small number of studies and/or a high within-study uncertainty relative to the between-study variation [23]. However, in this case, the results from the Cochran Q test suggest that a fixed-effects multivariate model may be preferable, and as expected, this model returns almost identical estimates for the pooled summary associations (results not shown).

**Figure 4:**
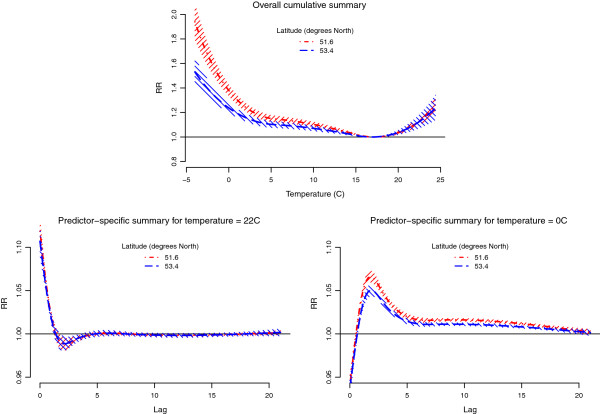
**Pooled temperature-mortality association by latitude in 10 regions of England and Wales, 1993–2006.** Predictions for the 25^th^ (dot-dashed line) and 75^th^ (dashed line) percentiles of latitude from meta-regression for overall cumulative summary (top panel), and predictor-specific summaries at 22°C (bottom-left panel) and 0°C (bottom-right panel). Reference at 17°C. The 95%CI are reported as shaded areas.

The heterogeneity across regions can be partly explained as effect modification by region-specific variables. The results of the example of meta-regression with latitude are illustrated in Figure 4. The top panel suggests a differential overall cumulative association between northern and southern regions, a pattern previously reported [8,10]. Interestingly, the effect modification seems to occur for cold, with a higher effect in southern regions, but not for heat. The estimated pooled RR at 0°C versus 17°C are 1.380 (95%CI: 1.337–1.424) and 1.237 (95%CI: 1.198–1.277) for the 25^th^ and 75^th^ percentiles of latitude, respectively, while the same estimates are 1.106 (95%CI: 1.079–1.133) and 1.104 (95%CI: 1.059–1.150) for 22°C. Overall, the evidence for an effect modification is substantial, with a highly significant Wald test (*p*-value < 0.001). Latitude explains much of the heterogeneity across regions, with an *I*^2^ reduced to 18.7% and a non-significant Cochran Q test (*p*-value = 0.174). The bottom panels illustrates the same effect modification for predictor-specific summary associations at 22°C and 0°C. Consistently, the Wald test indicates a significant effect for cold (*p*-value < 0.001), but not for heat (*p*-value = 0.634).

## Discussion

In this contribution we describe a method to re-express the bi-dimensional fit of DLNMs in terms of uni-dimensional summaries, involving reduced sets of modified parameters of the basis functions chosen for the space of predictor or lags. This development, in addition to simplifying the algebraic definition of the methodology, offers a more compact description of the bi-dimensional association modelled by DLNMs. In particular, the dimension of the sets of reduced parameters is usually compatible with the application of multivariate meta-analytical techniques in a two-stage framework, allowing the analysis of complex non-linear and delayed associations in multi-location studies.

Previous applications of the two-stage design for multi-location time series studies are based on simplified functions for modelling the association of interest at the first stage. In particular, the analyses are usually limited to splines or other non-linear functions of simple moving average of the exposure series [6-8], a modelling approach similar to the alternative models used for comparison in our example. Alternatively, the simplification could be applied in the other dimension of predictor, specifying DLMs for linear or linear-threshold exposure-response relationships [8-11]. All these approaches require strong assumptions on the exposure-response dependency, in order to simplify the association modelled in one of the two dimensions within the first stage. These are prone to biases when the true underlying dependency is misspecified. The framework we propose, in contrast, require less assumptions or simplifications regarding the association in the first-stage model, but rather reduces the estimates to uni-dimensional summaries of a bi-dimensional fit. The advantages of this approach are exemplified by the comparison of the simpler alternatives with the bi-dimensionally flexible model, illustrated in the Results section. This methodology offers greater flexibility in the investigation of complex associations through a two-stage analysis.

Most of the limitations of DLNMs and multivariate meta-analysis of multi-parameter associations, previously discussed [5,12], identically apply to this framework. In particular, the issues of model selection and control for confounding pose important challenges, and are matters of current and future research. The issue of model selection is particularly relevant, due to the bi-dimensional nature of the models, where two independent bases are chosen to describe the dependency along predictor and lag spaces, respectively. In our example, we selected the bases a-priori for illustrative purposes, but model selection is clearly more problematic in applied analyses.

The problem of estimating perfectly correlated random components in the second-stage meta-analytical model, as described in the example, can bias upward the standard errors of the pooled estimates. This problem occurs in likelihood-based and method of moments estimation procedures of multivariate meta-analysis, as these estimators truncate the between-study correlations on the boundary of their parameter space [16,19,23]. Although in many cases this problem arises with small number of studies or when the amount of heterogeneity is negligible (and thus when a fixed-effects model is preferable), alternative approaches may be considered. First, different estimation methods can be applied, for example by imposing some structure to the between-study (co)variance matrix, or adopting a Bayesian approach that employ weakly informative priors models could avoid truncation of between-study correlations. Also, alternative parameterization of the cross-basis functions may reduce the correlation pattern in the first stage and avoid estimation problems in the second-stage multivariate model. This issue needs to be explored further.

The definition of identical cross-basis functions in all the locations can be problematic in the presence of substantially different exposure ranges. In our example, the temperature distribution was similar across regions, and the placements of common knots was straightforward. However, this can be hardly generalized. The issue was previously discussed, and an alternative approach based on relative scale was proposed for pooling one-dimensional functions [12]. The same method is applicable for bi-dimensional DLNMs. However, this limitation requires further research.

Estimation methods for DLNMs not requiring the completely parametric approach proposed here seems attractive and possible, in particular based on penalized likelihood [24] or Bayesian methods [25]. These estimation procedures also provide automatic selection methods. These options require the specification of a large number of parameters, which are then shrunk during the fitting procedures to reach a far smaller number of *equivalent df*. However, the high dimensionality of the fitted model may present a problem for the second-stage multivariate meta-analysis, even when reduced to uni-dimensional summaries following (4)–(5). Techniques for meta-analysis of high-dimensional estimates are a topic of current and future research.

Potentially, the number of parameters of the second-stage multivariate meta-analysis can also be decreased by structuring the between-study (co)variance matrix of random effects. However, the extent to which such a choice can bias the estimates of fixed-effects parameters is not known. Moreover, this option is not yet available in the R package mvmeta, and will be implemented and assessed in future analyses.

## Conclusions

The extension of the DLNM framework presented here, involving the reduction of the complex two-dimensional fit to one-dimensional summaries, provides an improved method to study complex non-linear and delayed associations in two-stage analyses. Unlike previous approaches proposed so far, this method requires less simplification of the exposure-response shape or lag structure. This framework may be applied in any setting where non-linear and delayed relationships needs to be investigated in different populations or groups.

## Abbreviations

DLM: distributed lag model.

## Competing interests

The authors declare that they have no competing interests.

## Author’s contributions

BA firstly conceived the idea of re-expressing summaries of DLNMs in terms of one-dimensional functions. AG then derived the algebraic expression. AG and BA contributed to the structure of the manuscript and the design of the analysis in the examples. AG implemented the methodology in the R software, performed the analysis, and took the lead in drafting the manuscript. BA contributed to drafting the manuscript. All authors read and approved the final version of the manuscript.

## Pre-publication history

The pre-publication history for this paper can be accessed here:

http://www.biomedcentral.com/1471-2288/13/1/prepub

## Supplementary Material

Additional file 1 **Online appendix.** This pdf document provides additional information on the algebraic notation, on the software and R code, and on the time series data used in the example. Click here for file

Additional file 2 **Data.** This csv file includes the time series data for the 10 regions of England and Wales during the period 1993–2006, used in the example. Click here for file

Additional file 3 **R scripts.** This.zip file contains 6 R scripts. These are files with extension.R which can be used to reproduce the results of the analysis in the example. They can be opened directly in R or read with a text editor. Click here for file
