# Supplementary material for: Reducing and meta-analysing estimates from distributed lag non-linear models
Source: BMC Med Res Methodol. 2013 Jan 9;13:1. doi: 10.1186/1471-2288-13-1 (PMC3599933; doi:10.1186/1471-2288-13-1)
Supplement: Additional file 1 — Online appendix. This pdf document provides additional information on the algebraic notation, on the software and R code, and on the time series data used in the example. [file 1471-2288-13-1-S1.pdf]

Reducing and meta-analyzing estimates from distributed lag non-linear models – Supplementary online material

Antonio Gasparrini*[1] and Ben Armstrong[2]

[1]Department of Medical Statistics, London School of Hygiene and Tropical Medicine, London, UK
[2]Department of Social and Environmental Health, London School of Hygiene and Tropical Medicine, London, UK

Email: Antonio Gasparrini*- antonio.gasparrini@lshtm.co.uk; Ben Armstrong - ben armstrong@lshtm.co.uk;

*Corresponding author

This document is included as supplementary online material for the manuscript published in BMC Medical Research Methodology by Antonio Gasparrini and Ben Armstrong.

A Algebraic notation

We adopt a conventional notation to define a scalar x with an italic letter, a vector $\mathbf{x}$ with bold and a matrix $\mathbf{X}$ with capital bold. A three-dimensional array $\dot{\mathbf{X}}$ is indicated by the dot. Single indices as $\mathbf{X}_i$ refer to the object i in a list, while multiple indices in x_{ij} identifies the element in row i and column j of the matrix $\mathbf{X}$, with $\mathbf{x}_{i\cdot}^{\mathsf{T}}$ as the whole row vector. The index in square brackets in $\mathbf{x}_{[j]}$ or $\mathbf{X}_{[j]}$ indicates objects specialized from $\mathbf{x}$ or $\mathbf{X}$, while an index in round brackets refers to dimensionality of the object. In particular, $\mathbf{I}_{(i)}$ and $\mathbf{1}_{(j)}$ represent an identity matrix with dimension i and the j-length vector of 1's, respectively. The symbol $\otimes$ is used for Kronecker products.

B Software, code and data

B.1 Software and code

All the analysis and results described in this contribution are performed and produced with the R software, version 2.15.1 [1]. Distributed lag non-linear models are implemented in the package dlnm (version 1.6.4) [2], while multivariate meta-analysis and meta-regression techniques are performed using the package mvmeta (version 0.3.1) [3]. All the packages are available in the Comprehensive R Archive Network (CRAN) at http://www.R-project.org/ and downloadable through R.

The R code to entirely reproduce (and extend) the results illustrated in this paper is included as supplementary online material. The six scripts are used to perform the analysis and to reproduce all the results

described in the paper. The scripts are meant to be run consecutively.

B.2 Data

The data, including the daily series from the 1st of January 1993 to the 31st of December 2006, is supplied as the `regEngWales.csv` file in the online supplementary material. The same data were previously used in other publications [4, 5]. Latitude for each region in the multivariate meta-regression model was computed as the average of the latitudes of electoral districts (wards), weighted for the resident population. This information is inputted directly in the R script used for meta-regression.

Individual death record were obtained from the Office of National Statistics (www.ons.gov.uk). Records include date of death and postcode of residence at time of death. The postcode was used to divide deaths by government region and date to make daily series of counts for each region.

All available validated daily maximum and minimum dry-bulb temperatures and dew-point temperature were downloaded from the British Atmospheric Data Centre (badc.nerc.ac.uk). Daily mean temperature was estimated as the average of minimum and maximum. Daily mean relative humidity was computed from 9am and 3pm dew-point measurements, using the Clausius-Clapeyron equation. In preliminary validatory work on data from Heathrow airport stations, which reported complete hourly dew-point values, this summary was correlated at $r = 0.93$ in summer with the true 24-hour mean relative humidity.

For each measure we used only data from stations reporting on 75% of days between 1993-2006. This resulted in a mean of 29 stations contributing data to each regional series, from a minimum of 7 in London to a maximum of 44 in Wales. The temperature series were highly correlated within regions (mean $r = 0.96$, range 0.95–0.99) and station means varied little within region (mean $SD = 0.9°C$, range 0.4–1.01°C).

Finally, The data were processed to obtain population-weighted mean daily series for each region. The monitoring station series daily values were combined to make one mean series for each region incorporating two refinements:

1. to avoid noise in the regional aggregated mean series, missing values were first imputed from patterns in other stations in the region, using the method of the AIRGENE study [6];

2. daily mean values from these filled station series were calculated using weights equal to the populations residing closest to each station (*i.e.* within the Thiessen polygons surrounding the station).

References

1. R Development Core Team: *R: A Language and Environment for Statistical Computing*. R Foundation for Statistical Computing, Vienna, Austria 2012, [http://www.R-project.org/]. [ISBN 3-900051-07-0].
2. Gasparrini A: **Distributed lag linear and non-linear models in R: the package dlnm**. *Journal of Statistical Software* 2011, **43**(8):1–20.
3. Gasparrini A, Armstrong B, Kenward MG: **Multivariate meta-analysis for non-linear and other multi-parameter associations**. *Statistics in Medicine* 2012, **31**(29):3821–3839.
4. Armstrong BG, Chalabi Z, Fenn B, Hajat S, Kovats S, Milojevic A, Wilkinson P: **The association of mortality with high temperatures in a temperate climate: England and Wales**. *Journal of Epidemiology and Community Health* 2011, **65**(4):340–345.
5. Gasparrini A, Armstrong B, Kovats S, Wilkinson P: **The effect of high temperatures on cause-specific mortality in England and Wales**. *Occupational and Environmental Medicine* 2012, **69**:56–61.
6. Rückerl R, Greven S, Ljungman P, Aalto P, Antoniades C, Bellander T, Berglind N, Chrysohoou C, Forastiere F, Jacquemin Bea: **Air pollution and inflammation (interleukin-6, C-reactive protein, fibrinogen) in myocardial infarction survivors**. *Environmental Health Perspectives* 2007, **115**(7):1072–1080.
